# Predictive Value of Early Functional Improvement During Hospitalization for Intermediate‐Term Functional Outcomes After Stroke: A Retrospective Cohort Study

**DOI:** 10.1002/brb3.71858

**Published:** 2026-11-05

**Authors:** Qian Yu, Qinyi Gu, Jia Wang

**Affiliations:** ^1^ Department of Neurology Suzhou Hospital of Integrated Traditional Chinese and Western Medicine Suzhou Jiangsu China; ^2^ Department of Encephalopathy Suzhou Hospital of Integrated Traditional Chinese and Western Medicine Suzhou Jiangsu China; ^3^ Department of Spleen and Stomach Diseases Suzhou Hospital of Integrated Traditional Chinese and Western Medicine Suzhou Jiangsu China

**Keywords:** Barthel Index, early functional improvement status, functional change, National Institutes of Health Stroke Scale, stroke

## Abstract

**Objective:**

To investigate whether early functional improvement status is associated with 3‐month functional outcomes after stroke and to develop and internally validate a preliminary prediction model for poor outcomes.

**Methods:**

This retrospective cohort study included 150 patients with stroke who were hospitalized in the Department of Neurology and the Department of Rehabilitation Medicine at our hospital between March 2021 and December 2024 and who completed the 3‐month post‐discharge follow‐up. Early functional improvement was defined by changes in the Barthel Index and National Institutes of Health Stroke Scale (NIHSS) scores from admission to discharge. The cohort‐specific median values of ΔBarthel and ΔNIHSS were used as exploratory thresholds to classify patients into the functional improvement group and the insufficient functional improvement group. Baseline characteristics, early functional changes during hospitalization, and functional outcomes at 3 months after discharge were compared between the two groups. Poor functional outcome at 3 months after discharge, defined as a modified Rankin Scale score ≥3, was used as the dependent variable. Hierarchical multivariable logistic regression models were used to evaluate the association between early functional improvement status and poor functional outcome and its incremental prognostic contribution beyond baseline clinical variables. A nomogram was constructed based on the extended model. Model performance was evaluated using receiver operating characteristic curve analysis, calibration assessment, and decision curve analysis, and internal validation was performed using 1000 bootstrap resamples. In a sensitivity analysis, the categorized early functional improvement status variable was replaced by continuous ΔBarthel and ΔNIHSS values while retaining the same baseline covariates.

**Results:**

The functional improvement group was slightly younger than the insufficient functional improvement group. During hospitalization, the functional improvement group had lower NIHSS scores, higher Barthel Index scores at discharge, and greater changes in both functional measures from admission to discharge. At the 3‐month follow‐up, the functional improvement group had significantly higher Barthel Index scores, a higher rate of functional independence, and a lower incidence of poor functional outcomes (all *p* < 0.001). Multivariable analysis showed that age, admission blood glucose, and admission NIHSS score were inde‐pendently associated with higher odds of poor functional outcome, whereas admission Barthel Index and early functional improvement status were independently associated with lower odds of poor functional outcome. In the sensitivity analysis, each 1‐point increase in ΔBarthel was associated with lower odds of poor functional outcome (OR = 0.936, 95% CI: 0.898–0.977, *p* = 0.002), as was each 1‐point increase in ΔNIHSS (OR = 0.678, 95% CI: 0.524–0.878, *p* = 0.003). Adding early functional improvement status to the baseline clinical model modestly improved discrimination, with the apparent area under the curve (AUC) increasing from 0.809 to 0.852 (ΔAUC = 0.043, *p* = 0.037). After bootstrap correction, the AUC of the extended model was 0.827. These findings indicate modest incremental prognostic value within the present cohort, but external validation is required.

**Conclusion:**

Early functional improvement status during hospitalization after stroke was independently associated with intermediate‐term functional outcomes and may provide additional prognostic information beyond baseline clinical characteristics. The prediction model incorporating early functional improvement status and baseline clinical variables showed preliminary predictive performance within the present cohort but should not yet be regarded as a clinically validated risk‐stratification tool. External validation is required before its use in rehabilitation planning or clinical decision‐making.

## Graphical abstract

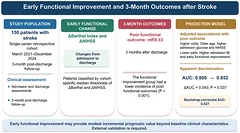

## 1 Introduction

Stroke is one of the leading causes of death and long‐term disability among adults worldwide. A substantial proportion of survivors are left with varying degrees of functional impairment, which significantly reduces quality of life and imposes considerable burdens on families and society (Feigin et al. 2025; Tang et al. 2024). Despite advances in acute‐phase management and rehabilitation strategies, functional recovery and intermediate‐term prognosis after stroke exhibit considerable inter‐individual variability. Even among patients with similar stroke subtypes, lesion locations, and initial severity, functional outcomes can differ markedly (Tani et al. 2025; Buvarp et al. 2023). Therefore, early identification of patients at risk of poor functional outcomes remains a critical issue in clinical management and rehabilitation decision‐making.

Previous studies have demonstrated that the severity of neurological deficits and baseline activities of daily living (ADL) at admission, as measured by the National Institutes of Health Stroke Scale (NIHSS) and the Barthel Index, are associated with stroke prognosis and are widely used for outcome prediction and risk stratification (Fu et al. 2024; Wu et al. 2024). In addition, some studies have begun to explore the potential impact of early rehabilitation interventions on functional recovery, suggesting that functional changes during hospitalization may be associated with subsequent outcomes (Otokita et al. 2021). However, most existing studies have relied on single‐time‐point assessments (Du et al. 2023).

The extent of functional improvement during hospitalization may vary considerably among patients with stroke (Lin et al. 2026). Relying solely on functional scores at admission or discharge cannot distinguish between patients with similar scores but different magnitudes of change between these two assessments, thereby limiting the prognostic information provided by a single assessment (Butsing et al. 2025). However, evidence regarding the prognostic value of early functional change assessed using routinely collected real‐world clinical data remains limited.

From a clinical perspective, hospitalization is an important period for monitoring early functional change and planning subsequent rehabilitation and follow‐up care (Wang et al. 2021). If early functional improvement status can be characterized using routinely collected functional assessment, it may provide supplementary information for identifying patients at increased risk of poor intermediate‐term outcomes and may help inform subsequent rehabilitation planning (Li et al. 2024). Therefore, investigating changes in functional status from admission to discharge and evaluating their prognostic significance are of considerable clinical and practical importance.

The present study therefore employed a retrospective cohort design using real‐world clinical data. Early post‐stroke functional improvement status was defined based on changes in the Barthel Index and NIHSS scores during hospitalization. The study examined whether early functional improvement status was independently associated with 3‐month functional outcomes, whether its addition to baseline clinical variables provided incremental prognostic information, and whether a preliminary prediction model incorporating this information showed adequate performance after internal validation.

## 2 Materials and Methods

### 2.1 Study Participants

This single‐center retrospective cohort study included 150 patients with stroke hospitalized in the Department of Neurology and the Department of Rehabilitation Medicine at our hospital between March 2021 and December 2024.

Inclusion criteria were: age ≥ 18 years; diagnosis of stroke confirmed by cranial computed tomography or magnetic resonance imaging; time from symptom onset to hospital admission ≤ 14 days; stable vital signs during hospitalization; complete documentation of functional assessments at admission and discharge in medical records; and completion of a 3‐month post‐discharge follow‐up.

Exclusion criteria were: severe cardiac, pulmonary, hepatic, or renal insufficiency; concomitant central nervous system diseases (e.g., Parkinson's disease or brain tumor); severe impairment of consciousness that precluded functional assessment; incomplete follow‐up data; or missing key clinical variables required for statistical analysis. The patient selection process is shown in Figure 1.

**FIGURE 1:**
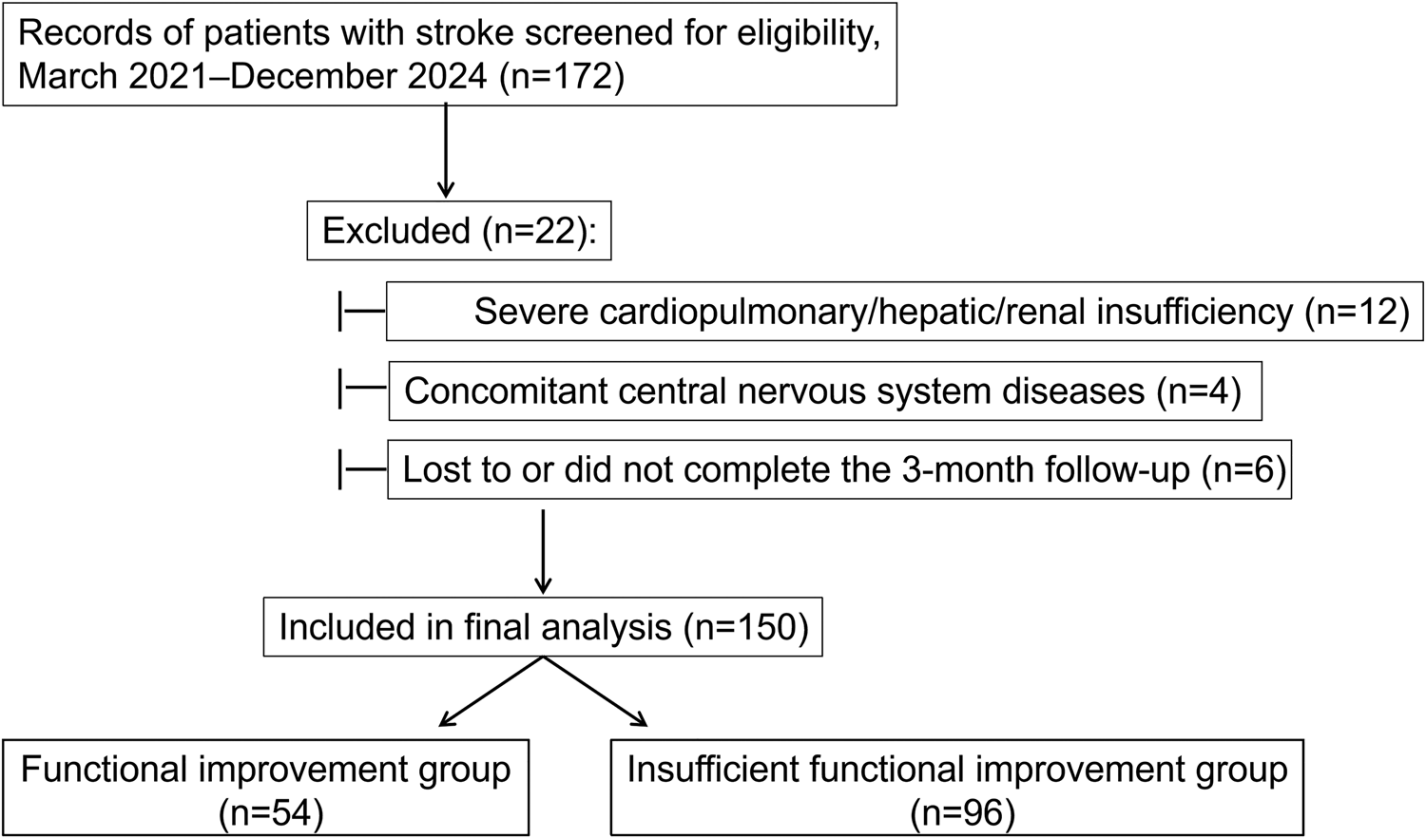
Flow diagram of patient screening, exclusion, and inclusion.

### 2.2 Study Procedures

Patient data were retrospectively extracted from the hospital electronic medical record system and nursing documentation, including data on routine supportive care and rehabilitation management received during hospitalization. All interventions were standard clinical care, and their content, timing, and intensity varied among patients.

During hospitalization, relevant nursing and rehabilitation care was provided by responsible nurses and rehabilitation therapists according to stroke subtype, disease severity, and functional status. Documented nursing and rehabilitation‐related content in the medical records included: (1) basic nursing and safety management: monitoring of vital signs, positioning management, and assessment and prevention of pressure ulcers and falls; (2) function‐related nursing and rehabilitation support: bedside activities, joint range‐of‐motion exercises, assisted sitting and ambulation training, and guidance on ADL (Wallace et al. 2007) according to the patient's functional status; (3) swallowing and nutritional support: assessment of dysphagia risk and provision of dietary guidance and nutritional support as indicated; and (4) post‐discharge follow‐up: the 3‐month functional outcome assessment was conducted through an outpatient visit or a telephone interview.

### 2.3 Definition and Classification of Early Functional Improvement Status

Early functional change during hospitalization was defined as the change in functional measures from hospital admission to discharge. This change was assessed retrospectively using the Barthel Index (Quinn et al. 2011) and NIHSS scores (Kasner et al. 1999) recorded at these two time points.

Functional change values were calculated as follows: Barthel Index change (ΔBarthel) = discharge Barthel Index − admission Barthel Index; NIHSS change (ΔNIHSS) = admission NIHSS − discharge NIHSS. Accordingly, higher values of both ΔBarthel and ΔNIHSS indicated greater functional improvement during hospitalization.

Patients with both ΔBarthel ≥ 25 points and ΔNIHSS ≥ 4 points were classified into the functional improvement group, whereas all other patients were classified into the insufficient functional improvement group. This classification was used to characterize early functional improvement status based on changes in the Barthel Index and NIHSS scores between admission and discharge. Because no established cutoff values exist, cohort‐specific median values of ΔBarthel and ΔNIHSS were used as exploratory thresholds.

### 2.4 Study Outcomes

Baseline demographic and clinical characteristics included age, sex, stroke subtype, stroke location, time from symptom onset to admission, history of previous stroke, comorbidities (hypertension, diabetes, and coronary heart disease), admission blood glucose, acute reperfusion therapy, admission NIHSS score, admission Barthel Index score, and length of hospital stay.

In‐hospital functional improvement indicators included discharge NIHSS score, discharge Barthel Index score, ΔNIHSS, and ΔBarthel. The NIHSS assessments at admission and discharge were performed by trained neurologists. The Barthel Index assessments at admission and discharge were conducted by trained rehabilitation therapists using standardized scoring procedures.

Intermediate‐term functional outcomes were obtained at the 3‐month post‐discharge follow‐up. The 3‐month modified Rankin Scale (mRS) assessment was obtained through an outpatient visit or a telephone interview. The primary outcome was the mRS score (Chye et al. 2022), which was analyzed as a binary variable: scores of 0–2 indicated a favorable functional outcome, whereas scores of ≥3 indicated a poor functional outcome. Secondary outcomes included the Barthel Index score at follow‐up and the rate of functional independence, with functional independence defined as a Barthel Index score of ≥90.

### 2.5 Statistical Analysis

All statistical analyses were performed using SPSS version 26.0 software (IBM Corp., Armonk, NY, USA) and R version 4.4.1 (R Foundation for Statistical Computing, Vienna, Austria). Continuous variables were tested for normality. Normally distributed variables were presented as mean ± standard deviation ($\bar{x}$ ± s) and compared using independent‐samples t‐tests. Non‐normally distributed variables were presented as median [M (P_25_, P_75_)] and compared using the Mann–Whitney U test. Categorical variables were expressed as number and percentage [n (%)] and compared using the χ^2^ test.

The dependent variable was the primary outcome defined above (mRS score ≥ 3). Univariate analyses were performed to examine the associations between candidate variables and the outcome. Candidate predictors for multivariable modeling were identified based on prior evidence regarding post‐stroke functional outcomes, clinical relevance, and their potential confounding effects. A hierarchical modeling strategy was used to evaluate the prognostic contribution of early functional improvement. The baseline clinical model (M0) included age, history of previous stroke, admission blood glucose, admission NIHSS score, and admission Barthel Index score. Early functional improvement status was subsequently added to M0 to construct the extended model (M1). The extended model was further adjusted for stroke subtype and acute reperfusion therapy in M2. Stroke subtype and acute reperfusion therapy were entered as categorical variables; no acute reperfusion therapy was used as the reference category for the latter. M0 was restricted to variables available at admission, whereas M1 was used to evaluate the additional prognostic information provided by early functional improvement status assessed at discharge. Because length of hospital stay defined the interval over which early functional change was measured and could influence the magnitude of the observed change, it was additionally included in a separate model (M3) to assess the robustness of the association. Associations were reported as odds ratios (ORs) with 95% confidence intervals (CIs). Variance inflation factors (VIFs) were calculated to assess multicollinearity among predictors. The incremental prognostic value of early functional improvement status was evaluated by comparing the baseline and extended models. Model discrimination was assessed using the area under the receiver operating characteristic curve (AUC), with differences evaluated using the paired DeLong test. Model fit was compared using the nested likelihood‐ratio test and Akaike information criterion (AIC). The Brier score was used to assess overall prediction error. Internal validation of the entire prediction model was performed using 1,000 bootstrap resamples. Bootstrap‐based optimism correction was applied to model discrimination and overall prediction error, yielding optimism‐corrected estimates of the AUC and Brier score. Model calibration was assessed using the calibration slope, calibration intercept, and bootstrap‐corrected calibration curve. Potential clinical utility was explored using decision curve analysis (DCA). A nomogram was constructed based on the extended model. To assess whether the findings depended on the median‐based categorization, a sensitivity analysis was performed by replacing the categorized early functional improvement status variable in M1 with continuous ΔBarthel and ΔNIHSS values, while retaining the same five baseline covariates: age, history of previous stroke, admission blood glucose, admission NIHSS score, and admission Barthel Index. ΔBarthel and ΔNIHSS were entered simultaneously into the sensitivity model. All statistical tests were two‐sided, and *p* < 0.05 was considered statistically significant.

## 3 Results

### 3.1 Comparison of Baseline Characteristics

Patients were categorized into the functional improvement group (*n* = 54) and the insufficient functional improvement group (*n* = 96). The functional improvement group was slightly younger than the insufficient functional improvement group (*t* = 2.29, *p* = 0.024). No significant differences were observed between the two groups in sex distribution, stroke subtype, stroke location, acute reperfusion therapy, history of previous stroke, hypertension, diabetes, or coronary heart disease (all *p* > 0.05). Time from symptom onset to admission was also similar between the two groups (*t* = 1.25, *p* = 0.21). At admission, NIHSS and Barthel Index scores were comparable between the two groups, with no statistically significant differences (NIHSS: *z* = 0.27, *p* = 0.78; Barthel Index: *z* = 1.33, *p* = 0.18) (Table 1).

**TABLE 1:** Comparison of baseline clinical characteristics between the two groups [$\bar{x}$ ± s, M (P_25_, P_75_), *n* (%)].

Characteristics	Functional improvement group (*n* = 54)	Insufficient functional improvement group (*n* = 96)	t/z/$x^{2}$	*P*
Age	61.63 ± 8.04	64.86 ± 8.48	2.29	0.024
Sex			0.59	0.44
Male	28 (51.85%)	56 (58.33%)		
Female	26 (48.15%)	40 (41.67%)		
Stroke subtype			1.43	0.23
Hemorrhagic	14 (25.93%)	34 (35.42%)		
Ischemic	40 (74.07%)	62 (64.58%)		
Stroke location			0.47	0.49
Anterior circulation	40 (74.07%)	66 (68.75%)		
Posterior circulation	14 (25.93%)	30 (31.25%)		
Acute reperfusion therapy			1.95	0.38
Intravenous thrombolysis	12 (22.22%)	15 (15.63%)		
Endovascular thrombectomy	4 (7.41%)	4 (4.17%)		
No reperfusion therapy	38 (70.37%)	77 (80.21%)		
History of previous stroke	12 (22.22%)	29 (30.21%)	1.11	0.29
Hypertension history			0.42	0.52
Yes	32 (59.26%)	62 (64.58%)		
No	22 (40.74%)	34 (35.42%)		
Diabetes history			0.52	0.47
Yes	16 (29.63%)	34 (35.42%)		
No	38 (70.37%)	62 (64.58%)		
Coronary heart disease history			0.17	0.68
Yes	14 (25.93%)	22 (22.92%)		
No	40 (74.07%)	74 (77.08%)		
Time from symptom onset to admission (*h*)	9.34 ± 3.95	10.07 ± 3.09	1.25	0.21
Admission blood glucose	7.35 ± 2.20	7.78 ± 2.05	1.21	0.23
Admission NIHSS score	9 (6.75, 11.25)	9 (7, 12)	0.27	0.78
Admission Barthel index	40 (28.75, 50)	40 (30, 50)	1.33	0.18
Length of hospital stay (*d*)	16.80 ± 5.30	17.90 ± 5.56	1.18	0.24

*Note*: NIHSS, National Institutes of Health Stroke Scale.

### 3.2 Comparison of In‐Hospital Functional Improvement

Compared with the insufficient functional improvement group, the functional improvement group had lower discharge NIHSS scores (*z* = 3.73, *p* < 0.001) and higher ΔNIHSS values (*z* = 6.89, *p* < 0.001). Similarly, the functional improvement group had higher discharge Barthel Index scores (*z* = 2.37, *p* = 0.018) and higher ΔBarthel values (*z* = 5.42, *p* < 0.001) (Table 2).

**TABLE 2:** Comparison of functional measures and changes from admission to discharge between the two groups [M (P_25_, P_75_)].

Characteristics	Functional improvement group (*n* = 54)	Insufficient functional improvement group (*n* = 96)	z	*P*
Discharge NIHSS score	3.5 (2, 6)	6 (3, 8)	3.73	<0.001
ΔNIHSS	5.5 (5, 6)	3 (2, 5)	6.89	<0.001
Discharge Barthel Index	70 (60, 80)	65 (50, 75)	2.37	0.018
ΔBarthel	32.5 (25, 40)	20 (11.25, 40)	5.42	<0.001

*Note*: ΔNIHSS was calculated as the admission NIHSS score minus the discharge NIHSS score; ΔBarthel was calculated as the discharge Barthel Index score minus the admission Barthel Index score. Higher values indicate greater functional improvement. NIHSS, National Institutes of Health Stroke Scale.

### 3.3 Comparison of Intermediate‐Term ADL Outcomes

At the 3‐month follow‐up, the functional improvement group had significantly higher Barthel Index scores than the insufficient functional improvement group (*z* = 4.75, *p* < 0.001). Moreover, the proportion of patients achieving functional independence (Barthel Index ≥ 90) was significantly higher in the functional improvement group (*χ* ^2^ = 20.66, *p* < 0.001) (Table 3).

**TABLE 3:** Comparison of intermediate‐term Barthel Index between the two groups [M (P_25_, P_75_), *n* (%)].

Characteristics	Functional improvement group (*n* = 54)	Insufficient functional improvement group (*n* = 96)	z/$x^{2}$	*P*
3‐month Barthel index	90 (83.75, 95)	80 (65, 90)	4.75	<0.001
Functional independence (Barthel index ≥ 90)	40 (74.07%)	34 (35.42%)	20.66	<0.001

### 3.4 Comparison of Poor Intermediate‐Term Functional Outcomes

At the 3‐month follow‐up, the functional improvement group had significantly lower mRS scores than the insufficient functional improvement group (*z* = 4.73, *p* < 0.001). Additionally, the proportion of patients experiencing poor functional outcomes (mRS score ≥ 3) was significantly higher in the insufficient functional improvement group than in the functional improvement group (*χ* ^2^ = 16.20, *p* < 0.001) (Table 4).

**TABLE 4:** Comparison of 3‐month functional outcomes between the two groups [M (P_25_, P_75_), *n* (%)].

Characteristics	Functional improvement group (*n* = 54)	Insufficient functional improvement group (*n* = 96)	z/$x^{2}$	*P*
3‐month mRS scores	2 (1, 2)	3 (2, 4)	4.73	<0.001
mRS score ≥ 3	11 (20.37%)	52 (54.17%)	16.20	<0.001

*Note*: mRS, modified Rankin Scale.

### 3.5 Multivariable Analysis of Early Functional Improvement Status and Poor Intermediate‐Term Functional Outcomes

In univariate analysis, patients with poor functional outcomes were older (*t* = 2.62, *p* = 0.01), were more likely to have a history of previous stroke (*χ* ^2^ = 4.60, *p* = 0.032), had higher admission blood glucose levels (*t* = 2.83, *p* = 0.005) and NIHSS scores (*z* = 3.63, *p* < 0.001), and had lower admission Barthel Index scores (*z* = 2.97, *p* = 0.003). Early functional improvement status was also significantly associated with 3‐month functional outcome (χ^2^ = 16.20, *p* < 0.001). No statistically significant associations with the outcome were observed for sex, stroke subtype, stroke location, acute reperfusion therapy, hypertension, diabetes, coronary heart disease, time from symptom onset to admission, or length of hospital stay (all *p* > 0.05) (Table 5).

**TABLE 5:** Univariate analysis of poor intermediate‐term functional outcomes [$\bar{x}$ ± s, M (P_25_, P_75_), *n* (%)].

Characteristics	Favorable functional outcome group (*n* = 87)	Poor functional outcome group (*n* = 63)	$x^{2}$/z	*P*
Age	62.20 ± 7.38	65.78 ± 9.39	2.62	0.01
Sex			0.18	0.67
Male	50 (57.47%)	34 (53.97%)		
Female	37 (42.53%)	29 (46.03%)		
Stroke subtype			2.95	0.09
Hemorrhagic	23 (26.44%)	25 (39.68%)		
Ischemic	64 (73.56%)	38 (60.32%)		
Stroke location			0.84	0.36
Anterior circulation	64 (73.56%)	42 (66.67%)		
Posterior circulation	23 (26.44%)	21 (33.33%)		
Acute reperfusion therapy			1.16	0.56
Intravenous thrombolysis	18 (20.69%)	9 (14.29%)		
Endovascular thrombectomy	5 (5.75%)	3 (4.76%)		
No reperfusion therapy	64 (73.56%)	51 (80.95%)		
History of previous stroke	18 (20.69%)	23 (36.51%)	4.60	0.032
Hypertension history			0.74	0.39
Yes	52 (59.77%)	42 (66.67%)		
No	35 (40.23%)	21 (33.33%)		
Diabetes history			1.11	0.29
Yes	26 (29.89%)	24 (38.1%)		
No	61 (70.11%)	39 (61.9%)		
Coronary heart disease history			0.12	0.73
Yes	20 (22.99%)	16 (25.4%)		
No	67 (77.01%)	47 (74.6%)		
Time from symptom onset to admission (*h*)	9.41 ± 3.48	10.36 ± 3.31	1.69	0.09
Admission blood glucose	7.22 ± 1.96	8.19 ± 2.20	2.83	0.005
Admission NIHSS score	8 (7, 11)	11 (7, 13)	3.63	< 0.001
Admission Barthel Index	40 (35, 55)	35 (25, 45)	2.97	0.003
Early functional improvement status			16.20	< 0.001
Functional improvement	43 (49.43%)	11 (17.46%)		
Insufficient functional improvement	44 (50.57%)	52 (82.54%)		
Length of hospital stay (*d*)	17.56 ± 5.45	17.42 ± 5.55	0.15	0.88

*Note*: NIHSS, National Institutes of Health Stroke Scale.

To evaluate the independent association between early functional improvement and poor 3‐month functional outcome, a hierarchical modeling strategy was adopted. Covariates were selected based on prior evidence, clinical relevance, and their potential confounding effects rather than solely on statistical significance in the univariate analyses. M0 included age, history of previous stroke, admission blood glucose, admission NIHSS score, and admission Barthel Index, representing baseline demographic characteristics, cerebrovascular history, metabolic status, neurological deficit severity, and functional status, respectively. In M0, older age (OR = 1.078, 95% CI: 1.026–1.132, *p* = 0.003), higher admission blood glucose (OR = 1.279, 95% CI: 1.059–1.544, *p* = 0.011), higher admission NIHSS score (OR = 1.281, 95% CI: 1.108–1.480, *p* < 0.001), and lower admission Barthel Index (OR = 0.943, 95% CI: 0.912–0.974, *p* < 0.001) were associated with poor functional outcome, whereas history of previous stroke did not reach statistical significance (*p* = 0.070). Early functional improvement status was subsequently added to M0 to construct M1. After adjustment for the five baseline clinical variables, early functional improvement remained independently associated with lower odds of poor functional outcome at 3 months (OR = 0.154, 95% CI: 0.058–0.405, *p* < 0.001). Age (OR = 1.065, 95% CI: 1.013–1.121, *p* = 0.014), admission blood glucose (OR = 1.238, 95% CI: 1.011–1.517, *p* = 0.039), admission NIHSS score (OR = 1.322, 95% CI: 1.130–1.547, *p* < 0.001), and admission Barthel Index (OR = 0.933, 95% CI: 0.900–0.966, *p* < 0.001) remained independently associated with 3‐month outcome, whereas history of previous stroke was not statistically significant (*p* = 0.125).

The association between early functional improvement and poor functional outcome remained generally consistent across the additional adjustment models. After further adjustment for stroke subtype and acute reperfusion therapy (M2), early functional improvement remained associated with lower odds of poor outcome (OR = 0.151, 95% CI: 0.056–0.403, *p* < 0.001). Similarly, in M3, which additionally adjusted M1 for length of hospital stay, the association remained materially unchanged (OR = 0.138, 95% CI: 0.051–0.373, *p* < 0.001). Length of hospital stay itself was not independently associated with poor functional outcome (OR = 0.948, 95% CI: 0.876–1.024, *p* = 0.175). These findings indicated that the association between early functional improvement and 3‐month functional outcome remained generally consistent after additional adjustment for stroke characteristics, reperfusion treatment, and duration of hospitalization (Table 6).

**TABLE 6:** Multivariable logistic regression models for poor 3‐month functional outcome.

Variables	M0 OR (95% CI)	*P*	M1 OR (95% CI)	*P*	M2 OR (95% CI)	*P*	M3 OR (95% CI)	*P*
Age	1.078 (1.026–1.132)	0.003	1.065 (1.013–1.121)	0.014	1.065 (1.012–1.121)	0.015	1.066 (1.013–1.121)	0.014
History of previous stroke	2.212 (0.938–5.214)	0.070	2.033 (0.821–5.034)	0.125	2.014 (0.814–4.983)	0.130	2.060 (0.827–5.133)	0.121
Admission blood glucose	1.279 (1.059–1.544)	0.011	1.238 (1.011–1.517)	0.039	1.243 (1.012–1.525)	0.038	1.256 (1.021–1.544)	0.031
Admission NIHSS score	1.281 (1.108–1.480)	<0.001	1.322 (1.130–1.547)	<0.001	1.328 (1.131–1.559)	<0.001	1.355 (1.151–1.596)	<0.001
Admission Barthel index	0.943 (0.912–0.974)	<0.001	0.933 (0.900–0.966)	<0.001	0.932 (0.899–0.966)	<0.001	0.933 (0.900–0.967)	<0.001
Early functional improvement	—	—	0.154 (0.058–0.405)	<0.001	0.151 (0.056–0.403)	<0.001	0.138 (0.051–0.373)	<0.001
Stroke subtype	—	—	—	—	1.020 (0.412–2.527)	0.966	—	—
Intravenous thrombolysis vs. no reperfusion	—	—	—	—	1.238 (0.410–3.734)	0.705	—	—
Endovascular thrombectomy vs. no reperfusion	—	—	—	—	0.848 (0.061–11.852)	0.902	—	—
Length of hospital stay, per day	—	—	—	—	—	—	0.948 (0.876–1.024)	0.175

*Note*: M0 included age, history of previous stroke, admission blood glucose, admission NIHSS score, and admission Barthel Index. M1 additionally included early functional improvement status. M2 additionally adjusted M1 for stroke subtype and acute reperfusion therapy. M3 additionally adjusted M1 for length of hospital stay. The outcome was coded as 0 = favorable functional outcome and 1 = poor functional outcome. Insufficient functional improvement, hemorrhagic stroke, and no reperfusion therapy were used as the corresponding reference categories. OR, odds ratio; CI, confidence interval; NIHSS, National Institutes of Health Stroke Scale.

### 3.6 Incremental Prognostic Value of Early Functional Improvement Status

Model comparison showed that adding early functional improvement modestly increased apparent discrimination. The apparent AUC of M0 was 0.809 (95% CI: 0.737–0.881), whereas that of M1 was 0.852 (95% CI: 0.790–0.914), corresponding to an absolute increase in AUC of 0.043 (95% CI: 0.003–0.083). The difference in discrimination was statistically significant according to the paired DeLong test (*p* = 0.037). The addition of early functional improvement also significantly improved overall model fit. The nested likelihood‐ratio test comparing M0 and M1 yielded χ^2^ = 16.779 with 1 degree of freedom (*p* < 0.001), and the AIC decreased from 169.81 for M0 to 155.03 for M1. At the optimal Youden cutoff, M0 had an apparent sensitivity of 68.25% and specificity of 85.06%, whereas M1 had an apparent sensitivity of 82.54% and specificity of 74.71%. Internal validation using 1000 bootstrap resamples showed modest optimism in both models. The optimism‐corrected AUC was 0.783 for M0 and 0.827 for M1. The optimism‐corrected Brier score decreased from 0.189 in M0 to 0.172 in M1. The corresponding optimism‐corrected calibration slopes were 0.875 and 0.864, with calibration intercepts of 0.014 and 0.012, respectively (Table 7 and Figure 2).

**TABLE 7:** Comparison of model performance between the baseline and extended models.

Performance measure	M0: Baseline clinical model	M1: M0 + early functional improvement	Difference	*p* value
Apparent AUC (95% CI)	0.809 (0.737–0.881)	0.852 (0.790–0.914)	0.043 (0.003–0.083)	0.037
Optimism‐corrected AUC	0.783	0.827	—	—
Apparent Brier score	0.173	0.154	—	—
Optimism‐corrected Brier score	0.189	0.172	—	—
Sensitivity (%)	68.25	82.54	—	—
Specificity (%)	85.06	74.71	—	—
Optimal probability cutoff	0.502	0.347	—	—
AIC	169.81	155.03	−14.78	—
Optimism‐corrected calibration slope	0.875	0.864	—	—
Optimism‐corrected calibration intercept	0.014	0.012	—	—
Nested likelihood‐ratio test	Reference	—	χ^2^ = 16.779, df = 1	< 0.001

*Note*: M0 included age, history of previous stroke, admission blood glucose, admission NIHSS score, and admission Barthel Index. M1 additionally included early functional improvement status. Internal validation was performed using 1000 bootstrap resamples. ΔAUC was calculated as AUC_M1 − AUC_M0. AUC, area under the receiver operating characteristic curve; CI, confidence interval; AIC, Akaike information criterion.

**FIGURE 2:**
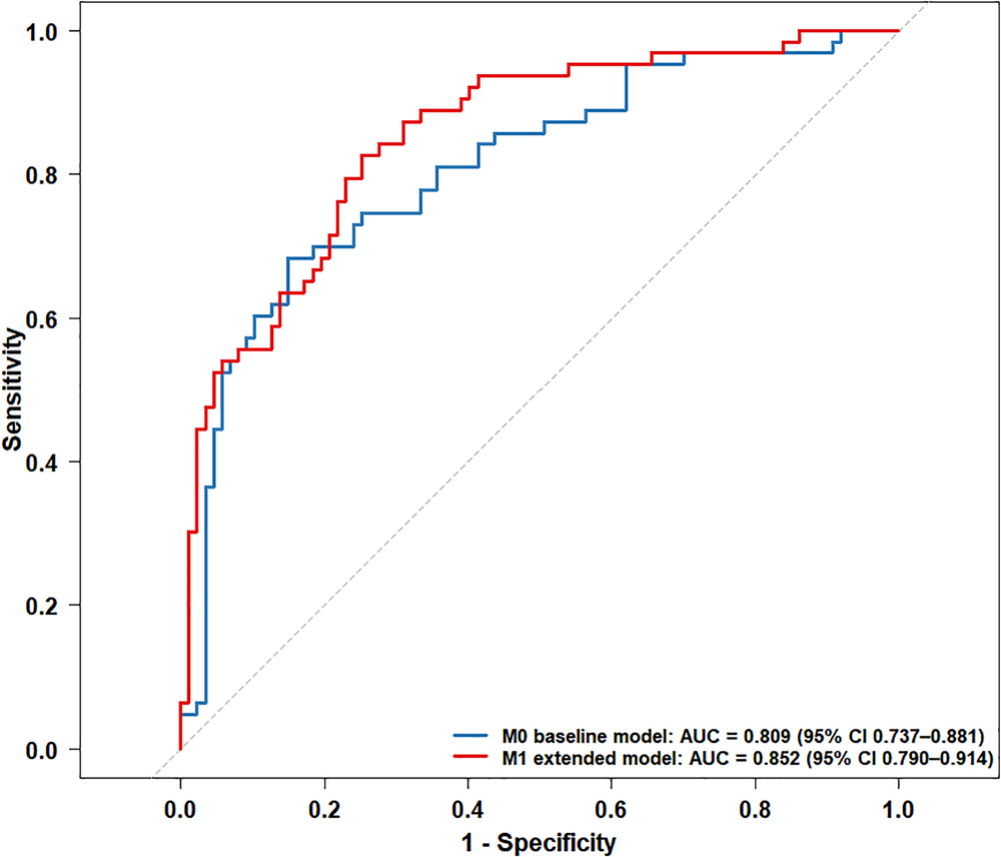
Receiver operating characteristic curves of the baseline clinical model (M0) and the extended prognostic model (M1) for predicting poor 3‐month functional outcome.

### 3.7 Sensitivity Analysis Using Continuous Functional Change Parameters

In the sensitivity analysis, each 1‐point increase in ΔBarthel was associated with lower odds of poor functional outcome at 3 months (OR = 0.936, 95% CI: 0.898–0.977, *p* = 0.002). Similarly, each 1‐point increase in ΔNIHSS was associated with lower odds of poor functional outcome (OR = 0.678, 95% CI: 0.524–0.878, *p* = 0.003). No substantial multicollinearity was observed among the variables included in the sensitivity model (VIF range: 1.024–1.078) (Table S1). These findings were consistent with the primary analysis based on categorized early functional improvement status.

### 3.8 Construction of the Nomogram for Predicting Poor Intermediate‐Term Functional Outcomes

Based on M1, a preliminary nomogram incorporating age, history of previous stroke, admission blood glucose, admission NIHSS score, admission Barthel Index, and early functional improvement status was constructed to estimate the probability of poor functional outcome at 3 months after stroke (Figure 3).

**FIGURE 3:**
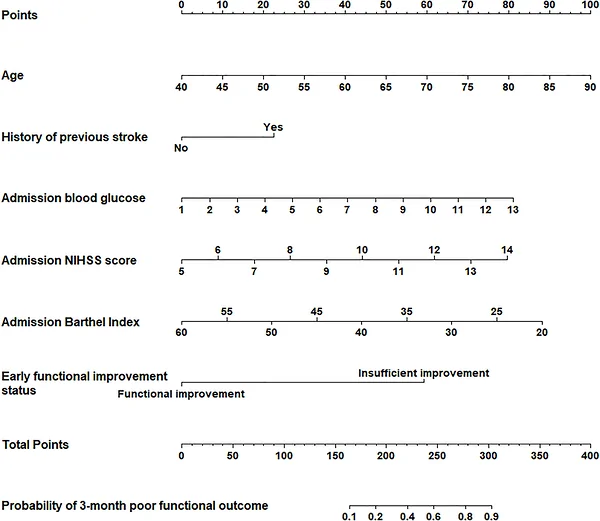
Nomogram based on the extended prognostic model (M1) for estimating the probability of poor 3‐month functional outcome after stroke.

### 3.9 Internal Validation and Calibration of the Extended Prognostic Model

Internal validation using 1000 bootstrap resamples showed that the bias‐corrected calibration curve of M1 remained reasonably close to the ideal reference line within the present cohort (Figure 4). The optimism‐corrected calibration slope was 0.864, with a calibration intercept of 0.012, indicating some degree of overfitting and limited systematic over‐ or underestimation within the development dataset. The mean absolute calibration error was 0.032, and the maximum absolute calibration error was 0.065. These calibration estimates require confirmation in an independent cohort.

**FIGURE 4:**
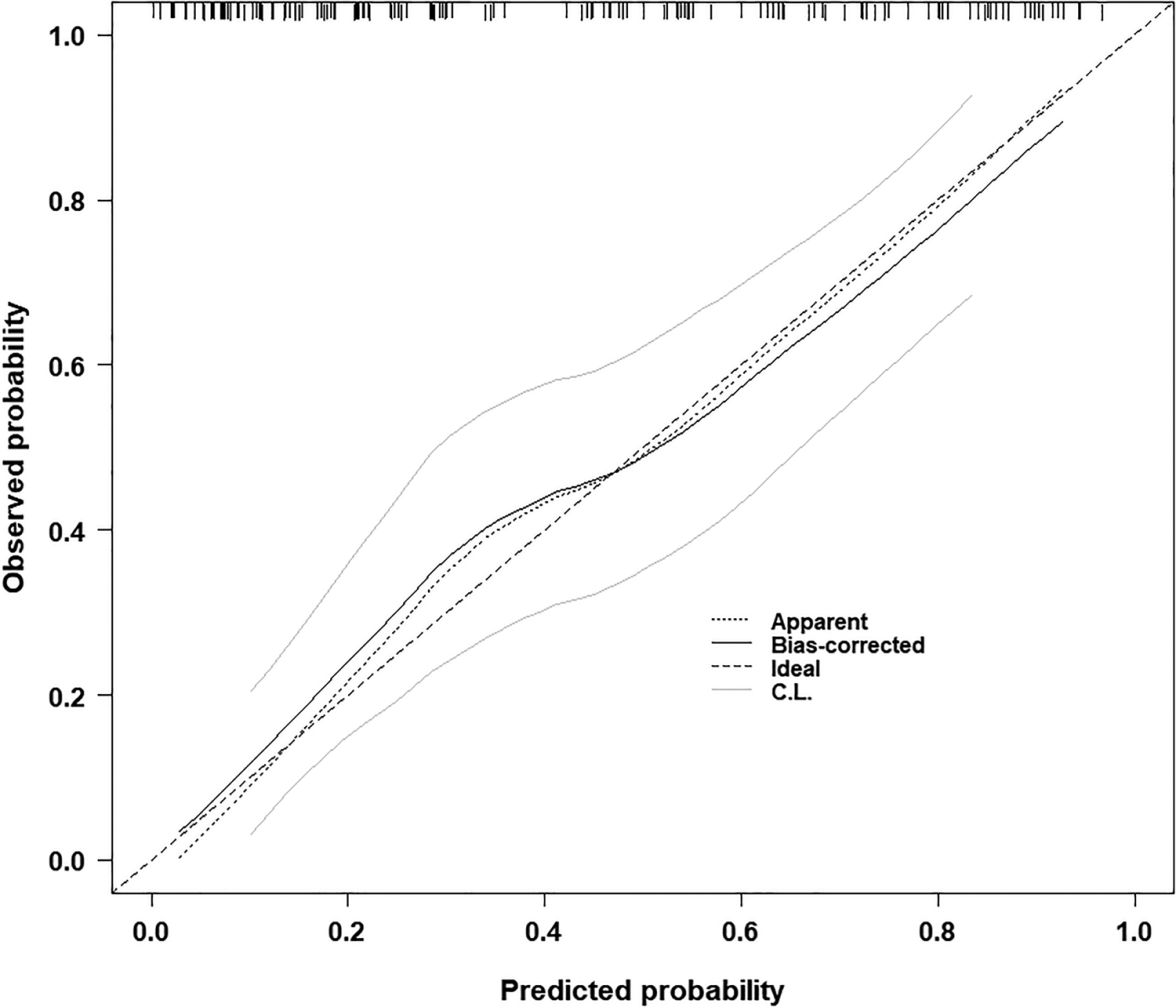
Bootstrap calibration curve of the extended prognostic model (M1) for predicting poor 3‐month functional outcome.

### 3.10 Clinical Utility of the Nomogram

Within the present cohort, DCA suggested that M1 provided a higher net benefit than the “Treat All” and “Treat None” strategies across portions of the evaluated threshold range (Figure 5). M1 showed a positive net benefit at threshold probabilities of approximately 0.05–0.85. Because the DCA was performed in the same relatively small cohort used for model development, these findings should be considered exploratory and do not establish the clinical utility of the model.

**FIGURE 5:**
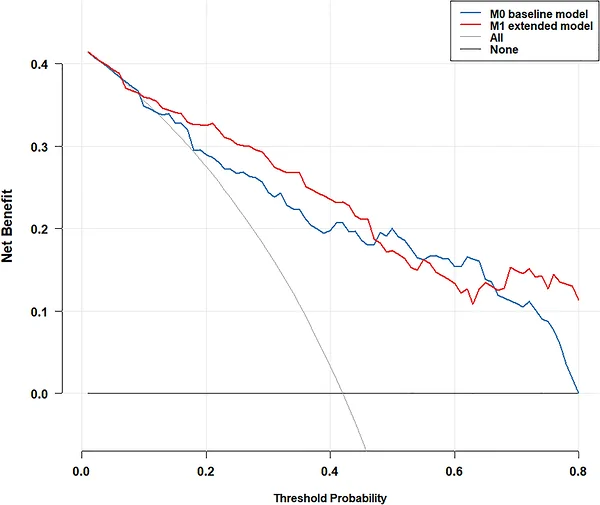
Decision curve analysis comparing the baseline clinical model (M0) and the extended prognostic model (M1) for predicting poor 3‐month functional outcome.

## 4 Discussion

This retrospective cohort study investigated the relationship between early functional improvement status during hospitalization and 3‐month functional outcomes in patients with stroke. The results showed that, even among patients with comparable neurological impairment and ADL at admission, substantial differences in early functional improvement were observed during hospitalization. Compared with patients with insufficient functional improvement, those in the functional improvement group achieved higher Barthel Index scores, lower mRS scores, and a lower incidence of poor functional outcomes at 3 months. After adjustment for age, history of previous stroke, admission blood glucose, admission NIHSS score, and admission Barthel Index, early functional improvement status remained independently associated with lower odds of poor functional outcome at 3 months. This association remained materially unchanged after additional adjustment for stroke subtype and acute reperfusion therapy and after accounting for length of hospital stay. These findings suggest that, beyond baseline functional status, early functional improvement during hospitalization may provide additional prognostic information regarding subsequent functional recovery after stroke.

Previous studies have consistently shown that admission NIHSS scores and Barthel Index can reflect acute stroke severity and baseline functional reserve and are associated with long‐term functional outcomes (Zhu et al. 2026). Our findings further corroborate this, indicating that higher NIHSS scores and lower Barthel Index scores at admission were significantly associated with poor intermediate‐term functional outcomes. However, it is important to note that these metrics primarily capture functional status at a single time point, representing an “initial level” rather than subsequent functional change (Fu et al. 2024). With advances in stroke care and rehabilitation concepts, increasing attention has been directed toward changes in post‐stroke functional status over time. Longitudinal studies using repeated assessments have shown that functional outcomes may depend not only on baseline status but also on subsequent changes during recovery (Lin et al. 2026).

In this context, our study extended the analytical perspective from single‐point functional scores to early functional change during hospitalization by constructing early functional improvement status based on combined changes in the Barthel Index and NIHSS. To assess whether the findings depended on the median‐based categorization, a sensitivity analysis was performed using continuous ΔBarthel and ΔNIHSS values. Higher values of both ΔBarthel and ΔNIHSS were associated with lower odds of poor functional outcome after adjustment for the same five baseline covariates. These results were consistent with the categorized analysis, suggesting that the main finding was not solely attributable to the selected thresholds or the dichotomization of functional changes.

Notably, our study found that early functional improvement status remained significantly associated with intermediate‐term outcomes after adjustment for age, history of previous stroke, admission blood glucose, admission NIHSS score, and admission Barthel Index. The association also remained stable after additional adjustment for stroke subtype and acute reperfusion therapy and in a separate model accounting for length of hospital stay. These findings suggest that the observed association was not fully accounted for by the measured baseline clinical variables, although residual confounding cannot be excluded because of the retrospective observational design (Krishnagopal et al. 2022). Previous studies have also reported that even among patients with similar stroke severity, subsequent functional changes may differ and be associated with later outcomes (Lin et al. 2026). Our findings suggest that changes in routinely available functional assessments between admission and discharge may provide supplementary prognostic information regarding subsequent functional outcomes. Beyond its association with 3‐month functional outcome, adding early functional improvement status to M0 to construct M1 was associated with a modest improvement in model performance. The apparent AUC increased from 0.809 to 0.852, corresponding to an absolute increase of 0.043, which was statistically significant according to the paired DeLong test. The significant likelihood‐ratio test and the decrease in AIC from 169.81 to 155.03 further supported improved model fit after early functional improvement status was added. After bootstrap correction, M1 retained a higher AUC and a lower Brier score than M0, suggesting that the observed improvement was not solely attributable to apparent performance in the original sample. Nevertheless, the magnitude of improvement in discrimination was modest, and the optimism‐corrected calibration slope of 0.864 indicated some degree of overfitting. Given the limited sample size and the number of predictors considered, M1 should be regarded as a preliminary prediction model. These results should therefore be interpreted as evidence of incremental prognostic value within the present cohort rather than proof of generalizable clinical performance.

From a clinical perspective, the findings may have potential relevance but require cautious interpretation. Hospitalization is an important period for monitoring early functional change and planning post‐discharge rehabilitation and follow‐up. In the present study, the nomogram incorporating early functional improvement status and baseline clinical variables showed moderate apparent discrimination and reasonably close calibration within the development cohort, while the exploratory DCA suggested potential net benefit across portions of the evaluated threshold range. At the optimal cutoff, adding early functional improvement status increased sensitivity from 68.25% to 82.54%, although specificity decreased from 85.06% to 74.71%. Within the present cohort, this trade‐off suggested improved sensitivity for identifying patients with poor outcome, but at the cost of a higher false‐positive rate. The clinical acceptability of this trade‐off requires evaluation in an independent cohort and in the intended clinical setting. The DCA results were also derived from the development cohort and should not be considered evidence of established clinical utility. However, M1 should be regarded as a preliminary prediction model rather than a clinically validated tool. External validation in an independent cohort is required to assess its discrimination, calibration, and clinical utility before any clinical application. If externally validated, early functional improvement status assessed using changes in routine functional measures between admission and discharge may help identify patients at increased risk of poor intermediate‐term outcomes and inform rehabilitation planning and follow‐up (Harari et al. 2020; Campagnini et al. 2025). This change‐based risk assessment may provide prognostic information beyond that obtained from a single‐time‐point evaluation.

However, this study has several limitations. First, it was a single‐center retrospective study with a relatively limited sample size, and the generalizability of the findings requires validation in multicenter cohorts and diverse populations. Second, the definition of early functional improvement status was based on functional assessments at admission and discharge, representing short‐term functional change during hospitalization rather than a complete longitudinal recovery trajectory. Future studies incorporating multiple assessment time points and trajectory modeling approaches are warranted. In addition, the thresholds used to define early functional improvement status were derived from the median values in the present cohort and may therefore be sample‐dependent. Although the sensitivity analysis using continuous ΔBarthel and ΔNIHSS values yielded results consistent with the categorized analysis, the reproducibility and clinical applicability of the classification require validation in independent cohorts. Third, although clinically relevant baseline and acute management factors were considered, detailed information on the timing, frequency, intensity, and duration of rehabilitation was not consistently available in the retrospective medical records. Consequently, differences in rehabilitation exposure between the two groups could not be adequately examined, and residual confounding related to rehabilitation or other unmeasured treatment factors cannot be excluded. The observed association should therefore not be interpreted as evidence that the recorded functional improvement was caused by a particular rehabilitation strategy. Future prospective studies with standardized rehabilitation records may help clarify the influence of these factors. Nevertheless, residual confounding related to rehabilitation exposure cannot be excluded. The observed association should therefore be interpreted cautiously, as it may partly reflect differences in rehabilitation exposure or other unmeasured treatment‐related factors. Fourth, imaging parameters, biomarkers, and other biological indicators were not included, which limited the exploration of potential mechanisms underlying early functional improvement. Future studies could integrate longitudinal functional assessments, imaging features, and biological indicators to refine early functional improvement assessment and explore its relationship with neuroplasticity mechanisms. Finally, the prediction model was developed and internally evaluated in the same relatively small, single‐center cohort. Given the sample size of 150 patients and the number of candidate predictors considered, overfitting cannot be excluded. Although internal validation using 1000 bootstrap resamples was performed to estimate and correct for optimism, bootstrap validation cannot replace evaluation in an independent population. Therefore, the model should be considered preliminary, and external validation is required to assess its transportability, calibration, and clinical utility before clinical application.

Overall, this study suggests that early functional improvement status during hospitalization is independently associated with intermediate‐term functional outcomes in patients with stroke and may provide incremental prognostic information beyond baseline clinical characteristics. These findings support further evaluation of change‐based functional measures in larger, externally validated cohorts before their use in rehabilitation planning or clinical risk stratification.

## 5 Conclusion

Early functional improvement status during hospitalization was independently associated with intermediate‐term functional outcomes at 3 months after discharge in patients with stroke. These findings suggest that early functional improvement status may provide supplementary prognostic information beyond baseline clinical characteristics. However, the prediction model remains preliminary and requires external validation before it can be considered for clinical risk stratification or rehabilitation planning.

## Author Contributions

**Qian Yu** conceived and designed the research study, acquired and analyzed the data, and wrote the initial draft of the manuscript. **Qinyi Gu** provided technical support for the acquisition and analysis of the data, performed statistical analysis, and contributed to the interpretation of the data and the manuscript revisions. **Jia Wang** provided expertise in the field of study, contributed to the interpretation of the data, and critically revised the manuscript for important intellectual content. All authors have read and approved the final manuscript, and have participated sufficiently in the work to take public responsibility for appropriate portions of the content.

## Funding

The authors have nothing to report.

## Ethics Statement

This retrospective study was conducted in accordance with the principles of the Declaration of Helsinki. The study protocol was approved by the Human Ethics Committee of Suzhou Hospital of Integrated Traditional Chinese and Western Medicine (Ethics Approval Number: 2025‐019). Given the retrospective nature of the study and the use of de‐identified clinical data, the requirement for informed consent was waived by the ethics committee.

## Conflicts of Interest

The authors declare no conflicts of interest.

## Supporting information




**Supplementary Table**: brb371858‐sup‐0001‐S1.docx

## Data Availability

The data used and/or analyzed during the current study are available from the corresponding author upon reasonable request.
