# Supplementary material for: Predictive Value of Early Functional Improvement During Hospitalization for Intermediate‐Term Functional Outcomes After Stroke: A Retrospective Cohort Study
Source: Brain Behav. 2026 Nov 5;16(11):e71858. doi: 10.1002/brb3.71858 (PMC13740360; doi:10.1002/brb3.71858)
Supplement: Supplementary file 1 [file BRB3-16-e71858-s001.docx]

**Supplementary Table S1**. Sensitivity analysis using continuous functional change parameters for poor 3-month functional outcome

| Variable | β | SE | Wald χ² | OR | 95% CI | *P* |
| --- | --- | --- | --- | --- | --- | --- |
| Age | 0.086 | 0.026 | 10.556 | 1.090 | 1.035–1.148 | 0.001 |
| Previous stroke | 0.789 | 0.470 | 2.825 | 2.202 | 0.877–5.528 | 0.093 |
| Admission blood glucose | 0.212 | 0.104 | 4.138 | 1.237 | 1.008–1.518 | 0.042 |
| Admission NIHSS score | 0.304 | 0.082 | 13.918 | 1.356 | 1.155–1.591 | < 0.001 |
| Admission Barthel Index | −0.081 | 0.019 | 17.506 | 0.922 | 0.888–0.958 | < 0.001 |
| ΔBarthel | −0.066 | 0.021 | 9.371 | 0.936 | 0.898–0.977 | 0.002 |
| ΔNIHSS | −0.388 | 0.132 | 8.679 | 0.678 | 0.524–0.878 | 0.003 |

Note: The outcome was coded as 0 = favorable functional outcome and 1 = poor functional outcome. In this sensitivity analysis, the categorized early functional improvement status variable in M1 was replaced by continuous ΔBarthel and ΔNIHSS values, while the same five baseline covariates were retained. All variables listed in the table were entered simultaneously into the multivariable logistic regression model. For previous stroke, “No” was used as the reference category. ΔBarthel was calculated as the Barthel Index at discharge minus that at admission, whereas ΔNIHSS was calculated as the NIHSS score at admission minus that at discharge; therefore, higher values of both measures indicate greater functional improvement during hospitalization. No substantial multicollinearity was detected (VIF range: 1.024–1.078). SE, standard error; OR, odds ratio; CI, confidence interval; NIHSS, National Institutes of Health Stroke Scale; VIF, variance inflation factor.
